# The knowledge norm of assertion: keep it simple

**DOI:** 10.1007/s11229-021-03362-7

**Published:** 2021-08-14

**Authors:** Max Lewis

**Affiliations:** University of Helsinki, Helsinki, Finland

**Keywords:** Norm of assertion, Express knowledge norm of assertion, Moral worth, Rightly/well distinction, Epistemic blame

## Abstract

The simple knowledge norm of assertion (SKNA) holds that one may (epistemically permissibly) assert that *p* only if one knows that *p*. Turri (Aust J Philos 89(1):37–45, 2011) and Williamson (Knowledge and its limits, Oxford University Press, Oxford, 2000) both argue that more is required for epistemically permissible assertion. In particular, they both think that the asserter must assert on the basis of her knowledge. Turri calls this the express knowledge norm of assertion (EKNA). I defend SKNA and argue against EKNA. First, I argue that EKNA faces counterexamples. Second, I argue that EKNA assumes an implausible view of permissibility on which an assertion is epistemically permissible only if it is made for a right reason, i.e., a reason that contributes to making it the case that it is epistemically permissible to make that assertion. However, the analogous view in other normative domains is both controversial and implausible. This is because it doesn’t make it possible for one to act or react rightly for the wrong reason. I suggest that proponents of EKNA have conflated requirements for φ-ing rightly (or permissibly) with requirements for φ-ing well. Finally, I argue that proponents of SKNA can explain the intuitive defectiveness of asserting on the basis of an epistemically bad reason (e.g., a random guess), even when the asserters know the content of their assertion, by arguing that the asserters are epistemically blameworthy.

## Introduction

According to the Simple Knowledge Norm of Assertion (SKNA): one may (epistemically permissibly) assert that *p* only if one knows that *p*. However, some argue that more is required for epistemically permissible assertion. Turri (2011) proposes the following amended view:

**The Express Knowledge Norm of Assertion (EKNA):** One may (epistemically permissibly) assert that *p* only if one’s assertion expresses one’s knowledge that *p*.^1^

To express one’s knowledge that *p* means that one’s knowledge that *p* non-deviantly causes one’s assertion.^2^

Williamson (2000), despite almost universally being interpreted as being a proponent of SKNA, seems to show sympathy for EKNA. He writes:

[I]f I know *p* and assert *p*, but the asserting is causally independent of the knowing, then something is wrong with the asserting. In some sense, in making my assertion I did not fully respect the knowledge rule [i.e., SKNA], although I was lucky enough to get away without violating it.^3^

It is understandable why both Turri and Williamson would endorse something like EKNA. After all, as we will see below, there is something seemingly defective happening when one knows that *p*, asserts that *p*, but there is no causal connection between one’s knowledge and one’s assertion.

My purpose in this paper is to defend SKNA. I do this in two parts. I argue that EKNA is false. I then argue that SKNA can meet two explanatory challenges suggested by EKNA. The first explanatory challenge is to account for the epistemic significance of asserting on the basis of one’s knowledge as opposed to merely asserting in accordance with one’s knowledge. The second explanatory challenge is to account for what is epistemically defective about asserting on the basis of epistemically bad reasons (e.g., random guesses).

The plan is this. First, I set the stage by clarifying key elements of my argument, i.e., the norm of assertion, epistemic permissibility, and epistemic blame. Second, I present trouble cases for SKNA from Turri (2011) and explain his proposed alternative (EKNA). Third, I argue that EKNA faces counterexamples. Fourth, I argue that EKNA assumes an implausible view of permissibility. On this view, an assertion is epistemically permissible only if it is made for a right reason, i.e., a reason that contributes to making it the case that it is epistemically permissible to make that assertion. However, the analogous view in other normative domains (e.g., morality, prudence, aesthetics, and rationality) is both controversial and implausible because it doesn’t allow for the possibility of φ-ing rightly (or permissibly) for the *wrong* reason. Fifth, I argue that while it is implausible that φ-ing for a right reason is necessary for φ-ing permissibly, it is plausible that it is necessary for φ-ing *well*. Sixth, I argue that SKNA is in a better position to accommodate the asserting rightly/asserting well distinction than EKNA. Finally, I argue that proponents of SKNA can explain the intuitive defectiveness of asserting on the basis of an epistemically bad reason (e.g., a random guess), even when the asserters know the content of their assertion, by arguing that the asserters are epistemically blameworthy. I conclude that we therefore ought to keep the knowledge norm of assertion simple.

## Setting the stage

Turri (2011), Williamson (2000), and many other philosophers interpret the norm of assertion as a *prescriptive* (or deontic) norm.^4^ Prescriptive norms concern what is permitted, required, or forbidden.^5^ In this sense, it is like certain moral, prudential, and aesthetic norms. For example, one may (morally permissibly) borrow someone else’s property only if you get their permission; one may (prudentially permissibly) move to Vienna only if that will contribute to one’s well-being; one may (aesthetically permissibly) create some work of art only if it is not kitsch.

Unlike these norms, the norm of assertion concerns what is *epistemically* permitted, required, or forbidden. That is, the source of the normativity is the epistemic domain, i.e., the normative domain concerned with truth, evidence, justification, knowledge, and so on. Moreover, the norm of assertion is independent of moral, prudential, and aesthetic prescriptive norms. This means that an assertion can be epistemically permissible (i.e., satisfy the norm of assertion) but not morally permissible. For example, one might assert a proposition one knows (and thus satisfy SKNA) but assert morally impermissibly, because it is cruel and hurts someone’s feelings. On the other hand, one might assert epistemically impermissibly, but morally permissibly, e.g., one might assert a false or unknown proposition because doing so is necessary for saving someone’s life.

The norm of assertion is a requirement for epistemically permissibly asserting. This means that if one asserts and fails to satisfy the norm of assertion, one asserts epistemically impermissibly. An epistemic requirement, like other normative requirements, is such that if one is responsible for violating it, then one is blameworthy. Importantly, though, because the requirement one violates is epistemic, one is *epistemically* blameworthy in the way that one would be *morally* blameworthy for being responsible for violating a moral requirement.

Exactly what distinguishes epistemic blame from moral blame is a matter of controversy.^6^ While I cannot defend it here, I’m inclined to think that epistemic blame consists in a kind of intellectual distancing of oneself from the blamee—at least with regard to matters related to what they are blameworthy for. This intellectual distancing can manifest in multiple ways. One can intellectually distance oneself from a person (in the relevant sense) by becoming less disposed to: (a) trust their testimony, (b) recommend that a third party seek out that person’s testimony (c) treat the fact that they believe something as a reason to believe it oneself, (d) engage in a joint inquiry with that person, and (e) treat a disagreement with that person as a reason to alter one’s credence in the contested proposition.^7^ All of these changes in one’s disposition will likely be confined to a certain range of propositions depending on the specific epistemic failure for which the person is responsible. For example, if the person believes in multiple conspiracy theories that conflict with the scientific consensus (e.g., concerning the link between vaccines and autism or whether COVID-19 vaccines have tracking chips in them), then one’s epistemically blaming them might consist in being less disposed to trust their testimony concerning questions of vaccine safety or the intentions of the government.

Importantly, what makes this blame epistemic in nature is not only the way that one distances oneself from the blamee, but the reason for which one does this. As I will discuss further in the penultimate section, one reason to one distance oneself from the blamee is the insufficient regard or concern they exhibit for epistemically*-*important considerations (e.g., truth, justification, evidence, knowledge, and so on). However, this is compatible with thinking that this person is a morally good or virtuous person or even that the behavior for which they are epistemically blameworthy doesn’t make them morally blameworthy. For example, we can imagine a case in which a person’s epistemically blameworthy behavior does not have any morally-important consequences. Perhaps my friend believes that all Fermat numbers are prime (as Fermat himself conjectured) even though I have shown him Euler’s proof that this is false. My friend’s false and unjustified belief has no morally-important consequence for the world and is not the product of any moral vice (e.g., pride). Yet, I can still epistemically blame him for holding this false and unjustified belief or for asserting it to other people.

By seeing that there is a way in which a person can be epistemically blameworthy without being morally blameworthy, one can see at least one way in which epistemically impermissible behavior (e.g., violating the norm of assertion) can differ from morally impermissibe behavior (e.g., stealing).^8^ We also have a better idea of what the norm of assertion, as a prescriptive norm concerning epistemic permissibility, entails.

## The knowledge norm of assertion: simple versus complex

The Simple Knowledge Norm of Assertion has struck many as the correct account of when one may (epistemically permissibly) assert that *p*.^9^ However, Turri (2011) argues that it cannot explain the intuitive epistemic defectiveness of cases like the following:

**Random Randy:** Suppose Randy remembers that the postal code of his first apartment was SW1A 1AA. Because remembering that *p* entails knowing that *p*, Randy knows the postal code of his first apartment was SW1A 1AA. However, this knowledge is non-occurrent. Neither his knowledge nor his evidence for it is presently conscious. Oddly, this remains so even as his partner turns and asks him what the postal code of his first apartment was. (Despite its oddity, this is surely possible.) Without actually trying to recall, Randy randomly guesses that the postal code of his first apartment was SW1A 1AA, and confidently expresses this random guess to his partner.

**Spiteful Spiro:** Spiro is a spiteful guy who relishes causing people emotional pain. Out of spite, he plans to tell Lois that her fiancé just died. Some time before he embarks on executing his plan, he receives a text message from a reliable informant reporting that Lois’s fiancé has indeed just died. So Spiro knows that the fiancé died. But this knowledge doesn’t motivate him in the least to tell Lois that her fiancé died. He goes ahead and tells her out of pure spite.^10^

Turri argues that both assertions are intuitively epistemically defective, but SKNA cannot explain why because both Randy and Spiro know the content of their assertions.

Turri is certainly right that there appears to be something epistemically defective about these assertions. However, exactly how much of a problem these cases are for SKNA depends on how one understands the norm of assertion. If the norm of assertion is meant to be the set of *all* the necessary conditions for epistemically permissible assertion, then these cases are counterexamples to SKNA.^11^ If, however, the norm of assertion is meant to be *a* (or the most important) necessary condition for epistemically permissible assertion, then these cases are not, strictly speaking, counterexamples. Although, they do provide proponents of SKNA with an explanatory burden. However, as I will argue later on (in Sect. 7), proponents of SKNA can meet this explanatory burden.

How does Turri think those sympathetic to SKNA should respond? He suggests that they should endorse the following account instead:

**The Express Knowledge Norm of Assertion (EKNA):** One may (epistemically permissibly) assert that *p* only if one’s assertion that *p* expresses one’s knowledge that *p*.^12^

The core idea is that one’s assertion that *p* must be based on one’s knowledge that *p*. In particular, Turri thinks that one’s knowledge that *p* must non-deviantly cause one’s assertion that *p*.^13^ EKNA can explain why Randy’s and Spiro’s assertions are epistemically defective: neither of them expresses their knowledge. They know what they assert, but this knowledge plays no role in why they assert.

## Counterexamples to the express knowledge norm

Despite the fact that EKNA can easily explain why the cases of Randy and Spiro are intuitively defective, it has its own problems. There are cases in which one’s assertion is intuitively epistemically permissible and non-defective, but one’s assertion does not express one’s knowledge. There are cases in which one asserts on the basis of great evidence that one takes to establish or show that *p*.

**Evidential Eddie:** Suppose that Eddie knows that 16 × 57 = 912 because he has recently been practicing mental math and he remembered this equation. However, this knowledge is not occurrent. Oddly, this remains so even as his partner turns and asks what 16 × 57 equals. (Despite its oddity, this is surely possible.) In order to answer his partner’s question, Eddie decides to do the mental math again and he has a very strong intellectual seeming that 16 × 57 = 912. On the basis of the fact that he knows he’s good at mental math and that he has a very strong intellectual seeming that 16 × 57 = 912, he asserts, “16 × 57 = 912.” That is, he asserts solely on the basis of his current evidence that 16 × 57 = 912 (and not on the basis of his knowledge).

**Evidential Emma:** Emma comes to know from her friend, Harriet, who is a world-renown herpetologist, that Pythons are non-venomous. Later that day, her partner asks her if she knows of any non-venomous snakes. She asserts, “Pythons are non-venomous” solely on the basis of the fact that Harriet told her that Pythons are non-venomous. That is, she asserts solely on the basis of her great evidence that Pythons are non-venomous (and not on the basis of her knowledge that Pythons are non-venomous).

In these cases, the speakers assert solely on the basis of their great evidence. Moreover, their knowledge does not play any causal role and so their assertions do not express their knowledge. According to EKNA, these assertions are epistemically impermissible. However, these assertions are not only intuitively epistemically permissible, they are also intuitively non-defective. In fact, they represent very common bases for assertion. We often tell people things not just because we know them (even though we do) but because we have great evidence.

Another kind of case of an intuitively epistemically permissible (and non-defective) assertion is one in which a person asserts that *p* not because they know that *p* but because they know that they know that *p*:

**Second-Order Sandy:** Sandy is at dinner with his wife and his friend Peter. Sandy remembers that it rained yesterday and thus knows that it rained yesterday, but, like Randy, his belief is not occurrent. Neither his knowledge nor the first-order evidence for his knowledge is conscious to him. His wife just got back into town from a business trip and she asks Sandy whether it rained yesterday. Sandy has trouble recalling and Peter says to him, “Sandy, you definitely know that it rained yesterday. We got stuck in the rain without an umbrella.” Sandy knows that Peter has an incredible memory and so forms the belief that he knows that he knows that it rained yesterday. Sandy then asserts, “Yes, it rained yesterday” to his wife on the basis of his knowledge that he knows that it rained yesterday. That is, Sandy’s knowledge that he knows that it rained yesterday non-deviantly causes him to assert that it rained yesterday. However, Sandy’s belief that it rained yesterday remains non-occurrent and plays no causal role in his assertion.

Intuitively, Sandy’s assertion is not only epistemically permissible, but completely non-defective. After all, he asserts on the basis of an epistemic position that is better than merely knowing that *p*, but also entails that he knows that *p*. However, EKNA entails that Sandy’s assertion is epistemically impermissible.

Perhaps the proponent of EKNA will object that Sandy’s knowledge that it rained yesterday played a non-deviant causal role in Sandy’s coming to know that he knows that it rained yesterday and thus EKNA was not violated by Sandy’s assertion. But this move will not work, because Sandy’s knowledge that he knows that it rained yesterday is based on Peter’s assertion that Sandy knows that it rained yesterday and Peter’s assertion is obviously not caused (in any sense) by *Sandy’s* knowledge that it rained yesterday.

One might worry that these cases are very close to cases from Lackey (2008), which she argues are counterexamples to SKNA.^14^ According to Lackey, all that is necessary for epistemically permissibly asserting that *p* is that one: (1) be in a position to reasonably believe that *p* and (2) that one assert that *p* at least in part because one is in this position (i.e., on the basis of one’s evidence that *p*). Lackey calls this The Reasonable to Believe Norm of Assertion (RTBNA).^15^ One might argue that what explains the permissibility of my cases is that the asserters satisfy RTBNA. To see the similarity, consider the following case from Lackey:

**Creationist Teacher:** Stella is a devoutly Christian fourth-grade teacher, and her religious beliefs are grounded in a deep faith that she has had since she was a very young child. Part of this faith includes a belief in the truth of creationism and, accordingly, a belief in the falsity of evolutionary theory. Despite this, Stella fully recognises that there is an overwhelming amount of scientific evidence against both of these beliefs. Indeed, she readily admits that she is not basing her own commitment to creationism on evidence at all but, rather, on the personal faith that she has in an all-powerful Creator. Because of this, Stella does not think that religion is something that she should impose on those around her, and this is especially true with respect to her fourth-grade students. Instead, she regards her duty as a teacher to include presenting material that is best supported by the available evidence, which clearly includes the truth of evolutionary theory. As a result, while presenting her biology lesson today, Stella asserts to her students, ‘‘Modern-day *Homo sapiens* evolved from *Homo erectus*,’’ though she herself neither believes nor knows this proposition to be true.^16^

Lackey argues that Stella’s assertion is epistemically permissible despite the fact that she doesn’t know the content of her assertion.

I do not think the similarity between Lackey’s case and my own is a problem. While I cannot offer a full criticism of Lackey’s case or view, I can point to at least one important difference between my cases above and hers.^17^ In particular, the asserter in Lackey’s case is open to a kind of criticism or blame that the asserters in my cases are not. Recall that in Lackey’s case Stella not only fails to believe what she asserts, she actually believes it is false. This entails that she doesn’t know what she asserts. In addition, Stella knows that she doesn’t believe what she’s asserting. It seems like if her students were to find out her epistemic situation concerning what she asserted, they could blame or criticize her by saying, “But you (know you) don’t even believe that!”, “But you think that’s false!”, or “You don’t know that!”.^18^ SKNA can easily explain why Stella seems worthy of blame or criticism for asserting what she does: she doesn’t know the content of her assertion and she *knows* that she doesn’t know it. However, RTBNA cannot explain the appropriateness of such criticism or blame. This is because Stella is in a position to reasonably believe what she asserts and she asserts it because she is in this position.

## The right reason thesis

EKNA is not only subject to counterexamples, it also entails a controversial and implausible view of permissibility. Recall that EKNA entails an agent’s assertion that *p* is epistemically permissible only if the agent asserts that *p* because she knows that *p*. More generally, this amounts to the claim that an agent’s assertion that *p* is epistemically permissible only if the agent asserts that *p* for a right reason, i.e., a reason that at least contributes to making it the case that it is epistemically permissible to assert that *p*. The analogous requirement in other normative domains is both controversial and implausible. To assess the analogous requirement in other normative domains, let us formulate the thesis (without specifying the normative domain) as follows:

**The Right Reason Thesis:** An agent’s φ-ing is permissible only if that agent φs for a right reason, i.e., a reason that at least contributes to making it the case that it is permissible to φ.

In this section, I will argue that this thesis is implausible in all other normative domains—including in the epistemic domain concerning beliefs. The idea is that there is a symmetry between different normative domains and so what holds for permissibility in all other normative domains ought to hold for permissibility in the epistemic domain. Therefore, if versions of the Right Reason Thesis are false in all other normative domains, we have strong reason to think the epistemic version of it is false as well.

The Right Reason Thesis entails that the reasons for which one asserts are relevant to the epistemic permissibility of one’s assertion and, more specifically, that asserting for a right reason is necessary for asserting epistemically permissibly.^19^ However, in the moral domain, defenders of the full range of normative ethical theories have denied and continue to deny both of these claims. For example, consequentialists such as Mill (1871/2015), Moore (1912/2005), and Sidgwick (1962)^20^; deontologists or non-consequentialists such as Ross (1930/1939), Thomson (1999), Kamm (2007), and Scanlon (2008)^21^; and even some virtue ethicists, such as Zagzebski (1996), Hursthouse (1999) and perhaps even Aristotle.^22^

Whether Kant denied the relevance of an agent’s reasons for action for the moral permissibility of an action is controversial.^23^ However, there is some evidence that in the *Groundwork* that he thinks at least sometimes an agent’s reasons for action don’t bear on the moral permissibility of an action or at least that acting for a right reason is not required for acting morally permissibly. Consider his example of two shopkeepers.^24^ The first shopkeeper always gives his customer the correct change, but only for the reason that it is the prudent thing to do. In particular, he gives the correct change because doing otherwise would damage his reputation and that would, in turn, hurt his business. The second shopkeeper, on the other hand, always gives the correct change for a reason that makes it right for him to give the correct change (e.g., it’s the honest thing to do). The first shopkeeper acted morally rightly, but he does so for the wrong reason. The second shopkeeper both acts rightly and does so for the right reason. Kant seems to allow that they both act permissibly, but only the second shopkeeper’s action has a certain kind of moral value, which he calls moral worth.^25^

What Kant’s shopkeepers show us is that it is plausible that a person can do the right thing (i.e., act permissibly) for the wrong reason. More specifically, these cases show us that a person can do the morally right thing *only* for the wrong reason.^26^ What does it take for a reason to be the “wrong” reason to act? As I will understand it, when an action or reaction is permissible, there are normative reasons that either justify or require that one act or react in that way.^27^ These are the *right* reasons to perform an action. Any other reason is the wrong reason to perform that action in the relevant sense. This is because these are not the reasons that make it permissible for one to act or react in the relevant way.

If it is true that one can act rightly for the wrong reason, then it looks like the reasons for which an agent acts are at least not always relevant to the moral permissibility of the action.^28^ If this is true, then the Right Reason Thesis looks false concerning morality. However, I will now argue that it looks plausible that one can act or react rightly, but for the wrong reason in all normative domains—including the epistemic domain concerning beliefs. If this is correct, then we have strong reason to think that the Right Reason Thesis is false in all normative domains and therefore that the Express Knowledge Norm of Assertion is false because it entails a version of the Right Reason Thesis.

Before looking at the other domains, it is important to note that it is plausible that one can do the right thing for the wrong reason at either the objective level or the subjective level. One can do the objectively right thing without doing it for the reasons that make it objectively right, i.e., for the wrong reasons. One can do the subjectively right thing (e.g., what one’s evidence suggests is the right thing to do) without doing it for the reasons that make it subjectively right (e.g., the evidence that suggests that the action is right), i.e., for the wrong reasons. In what follows, I will rarely explicitly distinguish between objective and subjective permissibility, but what I say can be read as applying equally to both.

In aesthetics, it’s plausible that an action can be aesthetically permissible, but one can perform this action for the wrong reasons. For example, imagine that including a plot twist in one’s novel is the aesthetically right thing to do (e.g., because it would make the novel more compelling or powerful). One could still act aesthetically permissibly by adding the plot twist even if one did so for the wrong reason (e.g., because one knows it will help sales).^29^

It looks like one can do the rational thing (i.e., the rationally permissible thing) for the wrong reason. For example, imagine that acting rationally consists in acting in accordance with one’s beliefs and desires. Imagine that one desires to drink some cold lemonade and one believes that there is some cold lemonade in the refrigerator. It looks like the rational thing to do is to go to the refrigerator to get lemonade. However, one could do this for the wrong reason, e.g., one could go to the refrigerator to get lemonade because one thinks doing so will annoy one’s spouse, but one doesn’t desire to annoy one’s spouse. Here one does the rationally permissible thing but for the wrong reason.

One can also do the prudent thing but do it for the wrong reason. Let’s grant the acting prudentially consists in doing the thing that makes one’s life go best. For example, imagine that moving to Vienna will make one’s life go best (e.g., because one was offered a great job there). One could still act prudentially permissibly by moving to Vienna even if one did so for the wrong reason (e.g., because one likes Viennese cafes).

Even in epistemology, there is a difference between believing the right thing and believing it for the right reason. On the objective level, believing the right thing plausibly amounts to believing the truth.^30^ But one can believe the truth for the wrong reasons, e.g., the reasons that do not make the believed proposition true. On the subjective level, believing the right thing seems to amount to believing what one’s evidence supports. But one can believe what one’s evidence supports for the wrong reasons, e.g., reasons other than one’s evidence. In fact, the distinction between believing what one’s evidence supports and believing it for the right reasons is just the distinction between *propositional* justification and *doxastic* justification (or *ex ante* and *ex post* justification).^31^

One might object that some philosophers do argue that an agent’s reasons for action are relevant to the moral permissibility of the agent’s action.^32^ This is true, but these authors argue for theses that are considerably more modest than the Right Reason Thesis.^33^ First, they argue that an agent’s reasons for acting are only *sometimes* relevant to the permissibility of an action. That is, they allow that in many cases, the reasons for which an agent acts do not affect the permissibility of the action.^34^ Second, some only argue that acting for a morally *bad* reason can make a difference to the moral permissibility of an action.^35^ A morally bad reason is a moral reason that counts *against* performing the action in question. For example, it might be morally impermissible to perform some action if one performs the action for the reason that it will cause an innocent person harm—even if there are moral reasons in favor of performing the action (e.g., it will prevent greater harm to other innocent people).^36^ Thus, these views allow for the possibility of at least sometimes doing the morally right (or permissibly) thing for the wrong reason.

Notice that these views are importantly different from the Right Reason Thesis. First, the Right Reason Thesis holds that the reason for which one φs *always* matters for whether φ-ing is permissible. Second, this thesis holds that one must φ for a *right* reason, i.e., a reason that contributes to making it the case that it is permissible to φ. Thus, it not only forbids φ-ing only on the basis of a bad reason, but also φ-ing only on the basis of a “neutral” reason. A neutral reason is one that doesn’t count for or against the permissibility of φ-ing. For example, in the shopkeeper case, the prudential shopkeeper gives the right changes because it will help his reputation. That some action will help his reputation doesn’t count for or against the *moral* permissibility of his action and thus is a morally neutral reason. Thus, the moral version of the Right Reason Thesis entails that the prudential shopkeeper acts morally *impermissible* because he fails to act for a right reason. However, the view that acting for a morally bad reason can sometimes be relevant to the moral permissibility of an action doesn’t forbid this kind of action. Rather, it says that as long as the action is morally permissible (independent of the reasons for action) and the agent doesn’t φ for a morally bad reason, the action is permissible. Thus, moral philosophers who have defended the relevance of reasons for action for acting morally permissibly only defend a view that is considerably weaker than the moral version of the Right Reason View.

## φ-ing rightly versus φ-ing well

In the previous section, I argued that it is implausible (in all normative domains) that φ-ing rightly requires one to φ for some set of right reasons. But now the question arises of what normative significance φ-ing for right reasons has. After all, it seems implausible to say that whether or not one φs for the right reason is normatively insignificant. Therefore, in this section, I will outline one way in which it is normatively significant whether one φs for a right reason.

In all normative domains, there is an intuitive difference between φ-ing rightly (or permissibly) and φ-ing well.^37^ To get a better sense of the distinction, it will be helpful to highlight some essential features of the distinction. First, it can be a mere accident that one acts rightly. For example, in the case of the prudential shopkeeper, it is sheer fortune (or accident) that what prudence requires is the same as what morality requires and so acting on the basis of prudence leads the shopkeeper to do the morally right thing. However, when one acts well, it is not mere fortune that one does so. This is because when one acts well, one acts for some or all of the right reasons.^38^

Second, when one acts wells, it reflects well on the agent, e.g., they might be praise- or credit-worthy for so acting, because of this non-accidentality. For example, the shopkeeper who gives the correct change because it is the honest thing to do seems to deserve at least some credit for doing the right thing while the shopkeeper who gives the correct change only for prudential reasons does not. Or, consider the case of two chess players. The first player doesn’t know how to play chess. He moves the pieces he does and in the order he does because he likes the way the board looks with those pieces in the places he puts them. As luck would have it, he plays the right moves and wins the game. Now imagine that the second player makes the same moves as the first and in the same order, but she makes them for the reasons that make them the right moves (e.g., they increase her likelihood of winning). It seems clear that the first player makes the right moves, but the second player not only makes the right moves, but plays well.

Before moving on to show how the rightly/well distinction bears on EKNA, let me make clear that all I have argued for is that φ-ing for some or all of the right reasons to φ is *one* necessary condition for φ-ing well. Exactly what else is required is a matter of debate. For example, in the literature on acting morally well (i.e., acting with moral worth), it is common to think that something more than merely acting for some or all of the right reasons is required for acting morally well.^39^

My purpose in drawing the rightly/well distinction is not to provide a general account of φ-ing well, but to point out the normative significance of acting for some set of right reasons. In the next section, I will show that applying the rightly/well distinction to assertion allows proponents of SKNA to argue against EKNA. In particular, they can use the distinction to show that proponents of EKNA will have trouble offering a plausible account of asserting epistemically well, but proponents of SKNA will not.

## Asserting rightly versus asserting well

It seems to me that EKNA either does not make room for the rightly/well distinction or it does so only by positing a very implausible view of what it takes to assert well. First, notice that EKNA says that an assertion is *permissible* only if one performs that assertion for a right reason (i.e., only if the action expresses one’s knowledge). But now notice that doing the objectively or subjectively right thing in the domains of morality, the epistemic, aesthetics, prudence, and rationality does not require φ-ing for a right reason. Second, it is plausible that whether one φs well in these domains does depend on the reasons for which one φs (e.g., it seems like a necessary condition for acting morally well). Third, in other normative domains, an agent can φ rightly in a way that does not reflect well on the agent (e.g., when an author includes a plot twist, but only because it will help sales). But satisfying EKNA does reflect well on the asserter because she asserts what she knows non-accidentally and this is because her knowledge non-deviantly causes her to assert. But given that EKNA requires that one assert for a particular reason and that one cannot satisfy EKNA in a way that does not reflect well on the agent, it looks like it collapses the distinction between asserting epistemically rightly (or permissibly) and asserting epistemically well.

The fact that EKNA collapses the rightly/well distinction also explains why it is possible to construct counterexamples to EKNA. Because it is false that epistemically permissible assertions require asserting on the basis of a particular reason (i.e., knowledge), it makes sense that they can assert epistemically permissibly on the basis of other reasons (e.g., great evidence or second-order knowledge).

One might object that there is a plausible view on which epistemically permissibly *believing* requires one to believe for some right reasons, i.e., the Knowledge Norm of Belief (KNB). According to KNB, one may (epistemically permissibly) believe that *p* only if one knows that *p*. And, in order to know that *p* must believe that *p* for the right reasons. Given that the norm of belief and the norm of assertion are both epistemic norms and there is a tight connection between believing and asserting, one might argue that if the norm of belief correctly ignores the rightly/well distinction, then it is not surprising that the norm of assertion does as well.^40^

Of course, not everyone is convinced that there is a tight connection between the norm of belief and the norm of assertion.^41^ One might also think that KNB is problematic precisely because it does not allow for the rightly/well distinction either.^42^ Finally, asserting is an action and no normative domains require acting for specific reasons in order for an action to be right or permissible.

Even if EKNA did make room for the rightly/well distinction, it is not clear what it would be to assert that *p* epistemically well if EKNA were true. If we assume that acting for a reason is causal, as Turri (2011) does, then the epistemically right assertion needs to be caused by the fact that makes it epistemically right. But then EKNA entails that asserting epistemically well requires the following: one asserts that *p* because one knows that *p because* (i.e., for the reason that) one asserts that *p* because one knows that *p*. But it is not possible for anyone to satisfy this condition. Why? Because, given that it’s a causal relation, the thing doing the causing must exist *before* the thing that is caused. But the fact that one asserts that *p* because one knows that *p* doesn’t exist before one asserts that *p* because one knows that *p*. So, it’s not even clear that EKNA can provide an account of what it takes to assert that *p* epistemically well.

However, even if proponents of EKNA could offer a plausible account of what it takes to assert epistemically well, proponents of SKNA can offer a more plausible account. This is because as long as asserting epistemically well requires asserting for (or on the basis of) a particular reason, EKNA’s account of asserting well will be committed to a controversial claim. In particular, it will be committed to the claim that one can act for a second-order reason (i.e., a reason to act (or refrain from acting) for a reason). That is, it will be committed to the claim that asserting well will require that one assert on the basis of one’s knowledge *on the basis of another reason*. This requires one to assert for reason R1 for reason R2 or to assert on the basis of reason R1 on the basis of reason R2. But some argue that this is impossible.^43^

However, proponents of SKNA can argue that asserting that *p* epistemically permissibly requires that one know that *p* and that asserting that *p* epistemically *well* requires asserting that *p* on the basis of one’s knowledge that *p*. In other words, proponents of SKNA can argue that EKNA is actually a partial or full account of asserting epistemically well. Thus, proponents of SKNA can argue that asserting for a right reason is epistemically significant without having to argue that it matters for epistemic permissibility.

## A remaining question

Proponents of SKNA still have to explain why it seems that Randy’s and Spiro’s assertions are epistemically defective despite being epistemically permissible on their account. I think this can be done by appealing to the notion of epistemic blameworthiness. The idea is that while Randy’s and Spiro’s respective assertions are epistemically permissible (because they satisfy SKNA), both Randy and Spiro are epistemically blameworthy. In this section, I will argue that they are epistemically blameworthy given a plausible account of epistemic blame and that one can be epistemically blameworthy even when one acts epistemically permissibly.

I cannot defend a complete picture of what is necessary and sufficient for being morally or epistemically blameworthy here. What I will do, however, is to sketch a condition that is relevant to whether a person is morally or epistemically blameworthy and argue that it can explain what is intuitively defective with Randy’s and Spiro’s cases. I will therefore assume that Randy and Spiro at least satisfy whatever other conditions are necessary for being blameworthy (e.g., being responsible for asserting as they do).

As I will argue, the defect is not in the assertions themselves, but in the asserters. I argue that, as many have argued, an agent can be morally blameworthy even if her action is morally permissible. I conclude that it’s therefore plausible that an asserter can be epistemically blameworthy even if her assertion is epistemically permissible. Therefore, the proponent of SKNA can plausibly argue that what is intuitively defective about Randy’s and Spiro’s assertions is not that they are epistemically impermissible, but the reasons for which the assertions are made make Randy and Spiro epistemically blameworthy.^44^

One plausible set of views of moral blameworthiness are so-called “quality of will” accounts.^45^ One’s quality of will is the worth of the “regard or concern one has for others (or oneself), and toward the relevance of moral considerations, as manifested in one’s conduct.”^46^ Whether one is morally blameworthy is, at least in part, a matter of whether one expresses or indicates an insufficient degree of regard or concern for people or morally-important considerations. As McKenna (2012) notes, “One’s good, ill, or indifferent will, so understood, might well be exhibited in the reasons for which one acts, the intention with which one acts, or the choices one makes.”^47^

A classic case of a person expressing an insufficient degree or lack of regard or concern is one in which they intentionally try to harm someone. For example, imagine that Edward tells Susan that he hates her because he knows that this will hurt her deeply. Edward is intuitively morally blameworthy for this action. Why? One plausible answer is that Edward acts on the basis of a morally bad reason, i.e., a reason that counts against the moral permissibility of an action. It seems to me that because he acts for this particular reason he expresses an insufficient degree or even lack of regard or concern for a morally-important consideration, i.e., Susan’s feelings.

Moreover, one can also fail to express a sufficient degree of regard or concern by failing to act on certain reasons. Consider the following case from McKenna (2012):

Casper intentionally cancels his weekend business plans, and he does so with the intention of arranging a golf outing with his buddies. The reasons for his making these plans include a desire to spend some time in the sun, talk with a few old friends, enjoy a challenging game, and forget about his burdens at the office. His intentions and the reasons figuring in them are innocuous. But suppose that in making these arrangements, he did not consider taking time to spend with his daughter, who has recently become quite ill.^48^

Casper’s reasons for canceling his weekend business plans seem, as McKenna notes, “morally innocuous.”^49^ However, Casper is still intuitively morally blameworthy. This is because, even though he doesn’t act for a morally bad reason, he nonetheless ignores, and thus shows a lack of regard or concern for, morally-important considerations, e.g., his daughter and her failing health. In particular, he failed to even see the fact that his daughter is quite ill as a reason to cancel his weekend business plans.

While I cannot provide a full defense of the claim here, I think a very similar story can be told about epistemic blameworthiness. One condition that is important for determining whether a person is epistemically blameworthy for forming a belief or for making an assertion is whether or not they failed to express or display sufficient regard or concern for *epistemically-*important considerations (e.g., truth, justification, evidence, knowledge, and so on). For example, imagine that Harry asserts something he knows to be false to Bryan because he knows it is false and he wants to deceive Bryan. In this case, Harry is asserting on the basis of an epistemically bad reason, i.e., a reason that counts against the epistemic permissibility of that assertion. This is analogous to Edward asserting something hurtful to Susan because he knows it will hurt her. Whether or not Harry is morally blameworthy for this assertion, he is epistemically blameworthy for making it because it expresses an insufficient degree (or lack of) regard or concern for an epistemically-important consideration, i.e., truth.

Randy’s and Spiro’s respective assertions are also epistemically blameworthy because the reasons for which they assert express—or at least indicate—an almost complete lack of regard or concern for epistemically-important considerations.^50^ Randy does not even reflect for a moment about whether he knows what he asserts, whether he has any evidence for it, whether it is *prima facie* plausible, and so on. Rather he just makes a random guess and asserts it. As Turri writes, “*Without actually trying to recall*, Randy randomly guesses....”^51^ And, despite the fact that Randy knows the content of his assertion, this knowledge plays no role in explaining why he asserted it. Like Casper, Randy ignored the normatively-important consideration he had to act. Casper ignored the fact that his daughter was quite ill and Randy ignored the fact that he knew the content of his assertion. It seems clear that Randy is not at all concerned with the truth, justifiability, or knowability of his assertion. It also seems clear that Randy therefore expressed or displayed insufficient (or lack of) regard or concern for the relevant epistemically-important considerations. Thus, Randy is epistemically blameworthy for making his assertion.

Like Randy, Spiro does not even reflect on whether there are epistemically-important considerations that count for or against making his assertion. In fact, Turri (2011) tells us that even after he comes to know the content of his intended assertion, this knowledge does not play any motivational role in explaining why he asserted what he did.^52^ Rather he asserts only because he wants to cause Lois emotional pain. It seems clear that Spiro is not at all concerned with the truth, justifiability, or knowability of his assertion. It also seems clear that he therefore expressed or displayed insufficient (or lack of) regard or concern for the relevant epistemically-important considerations. Thus, Spiro is also epistemically blameworthy for making his assertion.

The claim that Randy and Spiro are epistemically blameworthy is bolstered by the account of epistemic blame that I sketched above. Recall that, on this view, epistemic blame involves an intellectual distancing. This distancing manifests in being less disposed to trust the blamee’s testimony or to recommend their testimony to a third party—at least concerning the topics related to what they are epistemically blameworthy for. It seems plausible that Randy’s partner should reduce her disposition to trust what he says concerning his old addresses (e.g., the postal codes) and Lois should reduce her disposition to trust anything Spiro says that might cause her emotional pain.

My argument that Randy and Spiro are epistemically blameworthy in virtue of their deficient epistemic quality of will dovetails with work by Frankfurt (2005) on “bullshit” and Cassam (2018, 2019) on epistemic insouciance. Frankfurt argues that the essence of a bullshit assertion is that it is “produced without concern with the truth” and “the motive guiding and controlling [the bullshit assertion] is unconcerned with how the things about which [the bullshitter] speaks truly are.”^53^ My epistemic quality of will account can explain why bullshitters are epistemically blameworthy: they fail to show sufficient regard or concern for an epistemically-important consideration (i.e., the truth). Like the bullshitter, Randy and Spiro assert without a concern for the truth. However, they are worse, epistemically speaking, than the bullshitter because they seem to assert without a concern for anything of epistemic value, e.g., truth, evidence, justifiability, justified belief, or knowledge. After all, one might not be concerned with whether something is true, but still be concerned about whether it is plausible or epistemically justifiable to others.

One might interpret Randy and Spiro as having what Cassam (2018, 2019) calls epistemic insouciance. Epistemic insouciance involves “an indifference or lack of concern with respect to whether [one’s] claims are grounded in reality or the evidence.”^54^ More specifically, it is an attitude toward inquiry in which one sees the search for knowledge through investigation as a “tedious chore that doesn’t merit one’s full attention.”^55^ Moreover, it involves contempt for the truth and experts as well as a general nonchalance about finding answers to complex questions because these questions are seen as less complex than they actually are.^56^ While Randy and Spiro might be epistemically worse than Frankfurt’s bullshitter, they do not seem to be quite as epistemically bad as the insouciant person. This is because they do not seem to have either the insouciant’s contempt for the truth and inquiry or her cavalier attitude toward the difficulty of answering complex questions. Moreover, the epistemically insouciant person has a general attitude about inquiry, which affects what and how they assert. However, Randy’s and Spiro’s assertions need not be a manifestation of such an attitude. The lack of regard they showed for the epistemically-important considerations could have been “out of character” or a one-off incident.

The final piece of this defense is the claim that a person can be blameworthy for φ-ing even if φ-ing is permissible.^57^ With a quality of will in hand, it is clear why this is possible: one can do the right (or permissible) thing and even know what the right thing to do is and still show insufficient regard for the normatively-important considerations. For example, imagine that Peter sees a small child drowning in a shallow pond. He knows that it is morally permissible for him to jump into the water and rescue the child. He jumps into the water and rescues the child. However, the only reason he did this was to impress his love interest, Jane. Had Jane not been there to witness Peter’s “heroic” act, he wouldn’t have rescued the child. It was clearly morally permissible for Peter to save the child, but Peter is nonetheless blameworthy. Why? Because despite rescuing the child, he was completely unmoved by the morally-important considerations of the situation. He therefore exhibited a lack of regard or concern for what is morally important and is morally blameworthy.^58^

Given that it seems quite plausible that one can be morally blameworthy even when one acts morally permissibly, it should also be plausible that one can be epistemically blameworthy even when one’s assertion is epistemically permissible—at least given the assumption that there is a strong symmetry between normative domains. We can also see the plausibility of there being epistemically permissible cases in which an asserter is nonetheless epistemically blameworthy. Imagine that Peter sees a car accident at the corner of 36th and Spruce. He knows that there was a car accident at 36th and Spruce. In fact, his knowledge that there was a car accident at 36th and Spruce is occurrent. He calls emergency services and asserts, “There has been a car accident at 36th and Spruce.” However, the only reason he makes this assertion is that he wants his love interest, Jane, to believe that he is a concerned citizen. Had Jane not been there to witness Peter’s helpful act, he would’ve given emergency services the wrong address as a prank. It was clearly epistemically permissible for Peter to “There has been a car accident at 36th and Spruce,” but he is nonetheless epistemically blameworthy. This is because the reason for which he makes his assertion shows that he lacks sufficient regard or concern for the epistemically-important considerations of his situation.

To sum up: proponents of SKNA can explain why there is something intuitively defective with Randy’s and Spiro’s respective assertions. They can argue that these asserters are epistemically blameworthy because they exhibit insufficient regard or concern for epistemically-important considerations (e.g., truth, justification, and knowledge). Their assertions are similar to what Frankfurt calls “bullshit” and related to the kinds of assertions that an epistemically insouciant person would tend to make. Finally, I argued that one can be epistemically blameworthy without one’s assertion being epistemically impermissible.

## Conclusion

In this paper, I defended the Simple Knowledge Norm of Assertion (SKNA) against the Express Knowledge Norm of Assertion (EKNA). First, I argued that EKNA faces counterexamples in which it is intuitively epistemically permissible to assert something on the basis of great evidence or second-order knowledge. Second, I argued that EKNA assumes an implausible view of permissibility according to which an assertion is epistemically permissible only if it is made for a right reason. I argued that proponents of EKNA were right to think that asserting for a right reason is epistemically significant, but that they were wrong to think that it was relevant to epistemic permissibility. Instead, I suggested that asserting for a right reason is necessary for asserting epistemically well. And, proponents of SKNA can provide at least a partial account of asserting epistemically well by arguing that asserting on the basis of one’s knowledge is required for asserting well. Finally, I argued that proponents of SKNA can explain why assertions made on the basis of epistemically bad reasons are intuitively defective. In particular, they can argue that the asserters are epistemically blameworthy in virtue of failing to show sufficient regard or concern for epistemically-important considerations. I conclude that we therefore have good reason to keep the knowledge norm of assertion simple.

## Footnotes

1 Turri (2011, p. 41).

2 Turri (2011, p. 41, fn. 12).

3 Williamson (2000, p. 268, fn. 16). I thank an anonymous referee for drawing my attention to Williamson’s apparent support of EKNA.

4 For example: Williamson (2000), Turri (2011), McKinnon (2015), Kelp (2018), Simion (2018). See Mehta (2016) for an exception.

5 McHugh (2012, p. 9).

6 For defenses of specifically epistemic notions of blame, see: Brown (2020), Boult (2020), and Piovarchy (forthcoming).

7 Related ideas are defended by Boult (2020) and Kauppinen (2018, pp. 10–11). However, Kauppinen says that he is talking about holding someone epistemically accountable and not epistemically blaming them.

8 While I have offered what I think are some key features of epistemic permissibility, my main argument does not rely on these details. I suspect that what I argue in the rest of the paper will be compatible with any account of epistemic permissibility that is roughly analogous to accounts of permissibility in other normative domains. I thank an anonymous referee for suggesting that I say more about epistemic permissibility.

9 Proponents of SKNA include (amongst others): Unger (1975), Slote (2010), DeRose (2002), Sosa (2010), Mehta (2016), Kelp (2018), and Simion (2018). Williamson (2000) is normally included in this list as well, but as the quotation above indicates, it is not clear that he endorses SKNA.

10 Turri (2011, p. 41).

11 Simion (2018) interprets the norm of assertion this way (479–480).

12 Turri (2011, p. 41).

13 Turri (2011, p. 42, fn. 12).

14 I thank an anonymous referee for this worry.

15 Lackey (2008, p. 125).

16 Lackey (2008, p. 111).

17 For a more complete response to Lackey on behalf of the knowledge norm of assertion, see: Milić (2017).

18 Kelp (2018, p. 432) also notes this response is appropriate.

19 What I argue also applies to any norm of assertion that constitutes some form of the Right Reason Thesis. Such accounts include ones by Lackey (2008, p. 125), Pelling (2013, p. 294), McKinnon (2015, p. 52), and Gerken (2017, p. 157). I argue against these views in Lewis (manuscript).

20 Mill (1871/2015, pp. 131–132), Moore (1912/2005, pp. 94–98), Sidgwick (1962, p. 204). Generally, consequentialists deny the relevance of the motives to moral permissibility.

21 Ross (1930, pp. 4–6; 1939, Chapter 6); Thomson (1999, p. 517); Kamm (2007, p. 138), and Scanlon (2008, Chapters 1–3).

22 Aristotle *NE* 1105b.

23 Timmons (2002).

24 Kant *G.* 4:397.

25 Kant *G.* 4:398.

26 After all, one might perform an action for both a right reason (e.g., a reason that makes it permissible for one to act) and a wrong reason.

27 For the distinction between the justifying strength and the requiring strength of normative reasons, see Gert (2007).

28 The idea is that one either acts for the right reason(s) or the wrong reason(s). It’s always permissible to do the right thing for the right reasons. It’s at least sometimes permissible to do the right thing for the wrong reason. Therefore, the reasons for which one acts are not always relevant to the permissibility of one’s action.

29 For example see: Hills (2020).

30 For a defense of a version of this claim, see: Whiting (2020). See also: Schroeder (2018).

31 This distinction comes from Firth (1978) who distinguished between “propositional warrant” and “doxastic warrant.” Goldman (1979) makes the distinction in terms of “ex ante. justification” and “ex post justification.”.

32 Sverdlik (1996, p. 339; 2011, p. 16), Gorr (1999, p. 582), Slote (2001), Tadros (2011, p. 140), Wedgwood (2011), and Liao (2012).

33 Only Slote (2001) defends a thesis close to the Right Reason Thesis. However, on Slote’s view, *only* the agent’s motives matter for moral permissibility. EKNA is therefore not analogous to Slote’s view and both Turri (2011) and Williamson (2000) would not be open to changing their view to be fully analogous to Slote’s. Moreover, Slote’s view is criticized for not being able to distinguish between acting rightly and acting rightly for the wrong reason. For example, see: Jacobson (2002) and Brady (2004).

34 For example, Sverdlik (1996, p. 339; 2011, p. 16), Gorr (1999, p. 582), Tadros (2011, p. 140), Wedgwood (2011), and Liao (2012).

35 McMahan (2009), Tadros (2011), Wedgwood (2011), and Liao (2012).

36 Liao (2012, p. 716).

37 For a defense of the claim that this distinction exists across different normative domains, see: Schroeder (2018). In the moral domain, this is normally cashed out as the difference between one’s action being right and one’s action having “moral worth.” However, I prefer to talk about the difference between φ-ing rightly and φ-ing well because it sounds more natural in domains outside of morality. For example, while I think it is intuitive that one can make a move in chess well, it sounds odd to talk about a move having “chess worth.”

38 There is a debate amongst moral philosophers concerning exactly what can count as a right-*making* reason. Some argue that the fact that the act is right is itself a reason that makes it right for one to act while others deny this. Proponents of the first view include Lord (2018) and Johnson-King (2019). Philosophers who deny this view include: Arpaly (2003) and Arpaly and Schroeder (2014). I will remain neutral on this question.

39 Proponents of this claim include: Arpaly (2003), Markovits (2010), Arpaly and Schroeder (2014), Singh (2020), and Howard (forthcoming).

40 For example, see: Williamson (2000, pp. 255–256).

41 For example, see: Simion (2019a, 2019b).

42 For further objections to the Knowledge Norm of Belief, see: Schroeder (2018, pp. 207–208).

43 Whiting (2017) and Lewis (manuscript).

44 Turri (2011, p. 43) considers a related objection according to which the asserters are blameworthy because their assertions are unreasonable. However, his arguments against this view do not apply to the account of blameworthiness I defend.

45 This account of blameworthiness originates with Strawson (1962). Proponents of some version of this view include: Scanlon (2008, Chapter 4), McKenna (2012), Shoemaker (2013), and Arpaly and Schroeder (2014).

46 McKenna (2012, p. 59).

47 McKenna (2012, p. 59).

48 McKenna (2012, p. 59).

49 McKenna (2012, p. 59).

50 This account also explains why Stella in Lackey’s Creationist Teacher case is epistemically blameworthy, but less blameworthy than Randy and Spiro. She does show concern for one epistemically-important consideration (i.e., what the scientific evidence supports), but she shows a lack of regard for other epistemically-important considerations (i.e., truth and knowledge).

51 Turri (2011).

52 Turri (2011, p. 41).

53 Frankfurt (2005, pp. 47, 55).

54 Cassam (2019, p. 79).

55 Cassam (2019, p. 86).

56 Cassam (2019, pp. 85, 79).

57 See the following for defenses of this claim: Zimmerman (1997), Haji (1998, Chapter 8), Scanlon (2008, Chapter 2), Parfit (2011, Chapter 7), Khoury (2011), and Capes (2012).

58 For other cases of being morally blameworthy despite acting morally permissibly, see: Driver (1992), Zimmerman (1997), Haji (1998, Chapter 8), Scanlon (2008, Chapter 2), Parfit (2011, Chapter 7), Khoury (2011), and Capes (2012).
